# Integrating 360° behavior-orientated feedback in communication skills training for medical undergraduates: concept, acceptance and students’ self-ratings of communication competence

**DOI:** 10.1186/s12909-016-0792-0

**Published:** 2016-10-18

**Authors:** Cosima Engerer, Pascal O. Berberat, Andreas Dinkel, Baerbel Rudolph, Heribert Sattel, Alexander Wuensch

**Affiliations:** 1 TUM Medical Education Center, TUM School of Medicine, Klinikum rechts der Isar, Technical University of Munich, Ismaninger Strasse 22, 81675 Munich, Germany; 2 Department of Psychosomatic Medicine and Psychotherapy, Klinikum rechts der Isar, Technical University of Munich, Langerstrasse 3, 81675 Munich, Germany; 3 CIP-Tagesklinik, Private Clinic for Psychotherapy, Maria-Josepha-Strasse 4, 80802 Munich, Germany

**Keywords:** Communication skills training, CST, Feedback, Feed-forward, 360° feedback, Training concept, Medical students, Medical education

## Abstract

**Background:**

Feedback is considered a key didactic element in medical education, especially for teaching of communication skills. This study investigates the impact of a best evidence-based practice feedback concept within the context of communication skills training (CST). We evaluate this concept for acceptance and changes in students self-ratings of communication competence.

**Methods:**

Our CST integrating feedback process comprises a short theoretical introduction presenting standards for good communication and a constructive 360° feedback from three perspectives: feedback from peers, from standardized patients (SPs), and from a trainer. Feed-forward process was facilitated for documenting suggestions for improvements based on observable behaviors to maximize learning benefits. Our CST was applied to four groups of eight or nine students. We assessed the data on students’ acceptance using a 6-point scale ranging from very good (1) to poor (6), applied a forced choice question to rank didactic items, and assessed changes in student’ self-ratings of their communication competence on a 10-cm visual analogue scale (VAS).

**Results:**

Thirty-four medical undergraduates (82 % female, 18 % male) in their first clinical year, with an average age of 21.4 years (SD = 1.0), participated in the new training. The concept achieved high acceptance from good to very good: overall impression (*M* = 1.56), sufficient interaction for discussion (*M* = 1.15), and constructive learning atmosphere (*M* = 1.18). Specific elements, such as practical training with SPs (*M* = 1.18) and feedback by SPs (*M* = 1.12), showed highest acceptance. The forced choice ranking placed all feedback elements at the top of the list (feedback (FB) by SPs, rank 2; FB by trainer, rank 3; FB by colleagues, rank 4), whereas theoretical elements were at the bottom (theoretical introduction, rank 7; memory card, rank 9).

Overall, student self-ratings of communication competence significantly improved in nine of the ten communication items assessed by VAS and showed a pre-post effect size of *ES* = 0.74 on a global rating.

**Conclusions:**

This study demonstrates that the training concept based on 360° behavioral feedback was well accepted and generated significant changes in student self-ratings of their communication competence. Further research is needed to determine the effects on objective communication performance.

**Electronic supplementary material:**

The online version of this article (doi:10.1186/s12909-016-0792-0) contains supplementary material, which is available to authorized users.

## Background

The importance of feedback was described as a didactic element in the health professions hundreds of years ago [1]. Since then, feedback has been increasingly integrated into medical education curricula [2–5]. The Accreditation Council for Graduate Medical Education has summarized teaching strategies for communication skills training and has included feedback as an important didactic tool [6]. Hattie and Timperley [7] in their important work ‘The Power of Feedback’ emphasized the impact feedback can have on the learning process. In the present era of competence-based curricula with a focus on soft skills and professional development, feedback is considered more essential than ever [8, 9]. Providing effective feedback to students is crucial to the didactic process of communication skills training (CST) [10–13].

A contemporary definition of feedback in medical education is as follows: *‘Specific information about the comparison between a trainee’s observed performance and a standard, given with the intent to improve the trainee’s performance’* [5]. Applying this definition in the context of CST means that there should be (i) a standard of good communication [14] and (ii) an elaborated process of how to provide information on ways to improve performance.

(i) There are different approaches to defining a standard of good communication and operationalizing good communication. Bensing et al. [15] assessed the suggestions for good physician–patient communication of 258 lay people in 32 focus groups in four different European countries. Listening to patients, showing empathy and personal attention were especially mentioned. The authors note that many suggestions by lay people are consistent with research literature about the operationalization of good communication.
  A dominant approach to this issue is known as the SPIKES Protocol, initially developed to train oncologists to aid in discussions where they had to break bad news to patients [16]. The SPIKES acronym stands for six steps: **S**etting, Patient’s **P**erception, **I**nformation Need, **K**nowledge, Responding to Emotions with **E**mpathy, and **S**ummary. Each step emphasizes skills that target different aspects of the communication challenge: Step one promotes a safe and private consultation** s**etting with a minimum of disturbances, step two assesses the **p**atient’s **p**erception and step three assesses the patient’s **i**nformation needs before disclosing information. Step four, **k**nowledge, emphasizes skills to structure the information to be shared. Step five focuses on addressing **e**motions that emerge during the consultation; and step six equips either the physician or the patient with skills which to **s**ummarize the content of the consultation. The SPIKES Protocol provides a toolbox of patient-oriented, flexible communication skills. The model has subsequently been extended to cover other communication challenges, such as providing complex information during discussions about joining a clinical trial [17] and leading effective consultations in emotionally challenging tasks such as talking about the shift from curative to palliative care [18].
(ii) The process on how to provide feedback includes several aspects: Feedback includes a feed-forward process, i. e. giving specific advice on how to improve communication [19–21], relies on the different perceptions of the teacher and the student [22], the student’s attitude [23, 24], and the acceptance of receiving feedback [4], especially as 360° feedback points out to feedback from multiple sources. A randomized controlled study investigated the impact of feedback on performance and acceptance by students. Precise, behavior-orientated feedback improved performance, but it was not appreciated as much as imprecise praising feedback [23]. McIlwrick et al. [25] concluded in their review about feedback deficiencies that one of the most important things is the enhanced understanding of the feedback tool and that residents learn good and constructive examples from their faculty teachers. Quilligan [26] reflected on the general complexity of providing feedback and points out that feedback needs to be individual, descriptive, focused, limited to the amount of information a receiver can implement, and provided at the appropriate moment, ideally with a forewarning about the feedback. Bokken et al. [27] discussed the complexity of feedback in their review. They extracted these key points: Standardised Patients (SPs) could provide constructive feedback; offering a safe learning environment; beginning with student self-evaluations; relate feedback to the learning goal and standard; be interactive, specific and descriptive; focus on observable behavior; and provide feedback by being subjective and using ‘I’ statements [27]. Strategies also include starting with positive before negative feedback, limiting feedback to key points and providing feedback immediately after performance. Van de Ridder et al. [28] also discussed possible variables that influence the impact of feedback on learning and underlined the importance of the quality of a precise observation and a rating of the performance. Related to this, the use of 360° feedback has become a reliable and valid tool for assessing physician performance in practice [29].

Even though feedback is highly recommended and appears standard in communication skills training (e.g. Rider, 2006), empirical evidence on the effects of feedback in communication skills training seems lacking. There are several reviews, guidelines, and expert opinions on the impact of feedback; however, to our knowledge, there have not been controlled trials to investigate the possible effects of different forms of feedback on various outcomes. Therefore, we focused on investigating the feedback concept on communication skills training itself.

We build on the current literature and conceptualize a new training concept by integrating behavior-orientated feedback in communication skills training, which addresses feasible solutions for all listed challenges. In the context of such courses based on role-playing with SPs [30], our feedback concept consists of a short, theoretical introduction to set a clear standard for good communication in a consultation, provide information on constructive feedback and feedback rules [24], and the process of role-playing with SPs [30]. We integrated 360° feedback from four different perspectives: self-reflection, feedback from peers [31], from SPs [10], and from a trainer; and, finally, emphasized the feed-forward dimension [20, 21] by documenting suggestions for improvement and reinforcing statements based on observable behaviors to a maximum of three each. Kirkpatrick [32] defines four levels on how to evaluate training programs: Level one is to evaluate reactions to CST, such as participants’ opinions or satisfaction with the training. Level two evaluates knowledge or self-assessed competence. Level three assesses the actual change in behavior. Level four looks to assess patient outcomes.

The present work demonstrates our concept by integrating current literature and recommendations. We present its evaluation by the participants as well as the changes in the students’ feelings of communication competence, according to Kirkpatrik [32] level one and level two.

## Methods

The data reported here were collected in the context of a randomized controlled trial with a focus on the effectiveness of providing feedback in the CST of undergraduates.

### Setting

Our medical school lasts six years and is divided into three educational phases: two years of basic science, three years of clinical science and skills, and ends with a practical year that consists of 48 weeks of clinical electives in different clinical disciplines. The three years of structured clinical education contains a longitudinal curriculum for communication with specific mandatory classes each year. In the first clinical year the class on communication skills training is held three afternoons in total, and takes place in small groups.

### Training concept of the CST and feedback procedure

We integrated our training concept into the curriculum, first on a voluntarily basis. The overall learning goal was focused on basic clinical communication skills, including three main topics: (1) the start of a consultation and building a trusting relationship (first afternoon), (2) structuring a consultation (second afternoon) and (3) addressing patient emotions (third afternoon). Each afternoon was structured the same way: a short *theoretical introduction*, then *role-playing* with SPs and elaborated *feedback*.

In the theoretical introduction, we defined a standard of good communication focusing on a specific topic per teaching unit. Our theoretical model was based on SPIKES [16], which can be used not only for breaking bad news, but other content that already has been described herein [17, 18]. We focused on and identified the specific skills listed below, which were summarized and distributed on a memory card during the first lesion (see Additional file 1):

1. the appropriate beginning and ending of a conversation and the perception of the patient’s perspective;
2. the structuring of the conversation and the expression of a common conversational aim;
3. identifying emotions and offering emotional support and, finally, in all teaching units
4. the competent use of general communication techniques, such as clear wording, appropriate non-verbal communication, using suitable pausing, reinforcing questioning and checking patients understanding.

In the *role-play*, we ensured that every student acted in the physician role for approximately 20 min once during the course as a general physician and was asked to take patient history from someone previously unknown. We provided case-vignettes for SPs with common complaints of general physicians’ practice, such as headache, back pain, colds, high blood pressure, or foot injuries. Different SPs were trained to act similar characters: All SPs were trained to play their roles in a similar way, but were afforded the flexibility to respond to the communication style of the active student. While one student was active in the role-play, the other students had observation tasks. These tasks consisted of answering questions on four different issues and mirroring the key elements of good communication, as had been provided earlier so that all the students were fully engaged in each role-plaing session to help them sustainably internalize the communication standards [33].

Next, we carried out the 360° *feedback* process in a standardised order: First, the active student was asked by the trainer for self-reflection with possible perceived challenges or specific questions or needs. Next, the observing students provided feedback according to their monitoring tasks from the theoretical introduction and the memory card by making behavioral observations, which were evaluated as good and to give feed-forward information on how to improve communication skills based on their notes. After that the SP, who had left the room after the role play, entered again to give behavioral feedback from the patient perspective. Accordingly, the SP feedback could not influence the group discussion and vice versa. SPs were trained beforehand in providing behavioral feedback. SP training integrated the above cited key points and recommendations by Bokken et al. [27]. Finally, the trainer finished the feedback process by summarizing the key points of the feedback process and adding his or her expert opinion in a written synopsis. The trainers focused on observable behaviors for three good applied skills and for three skills, that could be improved. The reports were handed out to the performing student as an individualised, take-home message. At all levels, we ensured that the method of providing feedback was constructive and related to visible and modifiable behaviours.

### Recruitment, participants and trainers

CST is a mandatory course in the first clinical year at our medical school. The new concept was announced as a study to test a new educational concept and offered on a voluntary basis. We promoted the course in introductory lecture for first clinical year students and on student Internet platform. All students had to sign up for a CST, but were free to choose this course integrated in a study or to sign up for a CST as an experienced based course with minimal structured feedback (training as usual). None of the students were aware of the topic or content of the educational study. We collected data on four students groups of eight or nine students trained in our developed concepts. Four trainers (PB, AD, BR, AW), all experienced in CST and in providing feedback to medical students, carried out the new training concept.

### Outcome measures and time of assessment

Participants were asked to complete two questionnaires to (i) evaluate the workshop and (ii) to rate self-competence.

(i) The evaluation questionnaire was adapted to the content and set-up of the training. There were eight items on general didactic elements: practical relevance of topics, practice orientation, interesting didactic conditioning, sufficient interaction, constructive learning atmospheres, personal benefits, fulfilled expectations, and overall impressions. There were nine items on specific didactic elements: theoretical introduction, memory card, practical training with SPs, monitoring tasks, self-reflection, feedback from colleagues, feedback from SPs, feedback from the trainer, and personal feedback form. Participants were asked to complete the questionnaire after the workshop. Each item was rated on a 6-point scale from 1 ‘very good’ to 6 ‘insufficient’, according to the academic grading system in Germany, see also Table 1. Additionally, a forced choice ranking from 1 ‘very important’ to 9 ‘less important’ was used for the nine didactic elements; see also Table 2.

**Table 1:** Evaluation of CST - Acceptance by Students

General Didactics	Mean	SD
Practical relevance of topics	1.5	0.83
Practice orientation	1.5	0.86
Interesting didactic conditioning	1.76	0.86
Sufficient interaction	1.15	0.44
Constructive learning atmosphere	1.18	0.39
Personal profit	1.53	0.75
Fulfilled expectations	1.5	0.62
Overall impression	1.56	0.71
Specific Didactic Elements		
Theoretical introduction	2.32	0.77
Memory card	2.15	1.18
Practical training with standardized patients	1.18	0.46
Monitoring tasks	1.94	0.74
Self-reflection	1.76	0.83
Feedback from colleagues	1.5	0.90
Feedback from standardized patients	1.12	0.33
Feedback from trainer	1.26	0.67
Personal feedback form	1.94	1.15

6- point scale: 1 : “best” to 6 “least”

*SD* standard deviation

**Table 2:** Evaluation of CST: Ranking of Forced Choice Items

Item	Ranking
Practical training with standardized patients	1
Feedback from standardized patients	2
Feedback from trainer	3
Feedback from colleagues	4
Self-reflection	5
Monitoring tasks	6
Theoretical introduction	7
Personal feedback form	8
Memory card	9

Every item had to be ranked from 1 “very important” to 9 “less important”; Items were ranked in ascending order based on the mean rank generated from each item
(ii) Furthermore, we assessed the changes in the student self-ratings of their communication competence. Students responded based on different 10-cm visual analogue scales (VAS) concerning their perceived personal competence in (A) overall communication and (B) six key communication aspects: starting conversations, patient perception, structuring conversations, patient emotions, ending conversations, and communication skills, such as clear wording, appropriate non-verbal communication, using suitable pauses, reinforcing questioning, and checking patient understanding of the content conveyed. All items corresponded to the memory card. The questionnaire was completed before and after the CST, see Table 3.

**Table 3:** Student Self-ratings of Communication Competence

	Pre (SD)	Post (SD)	p*	ES^a^
Global Rating	5.48 (1.60)	6.62 (1.33)	0.001	0.74
Starting Conversations	5.55 (2.00)	8.68 (1.04)	0.000	1.94
Patients’ Perception	6.68 (1.55)	7.05 (1.20)	0.125	0.30
Structure of Conversation	4.97 (1.56)	6.23 (1.23)	0.000	0.89
Patients’ Emotions	6.39 (1.65)	7.22 (1.53)	0.001	0.52
Ending Conversations	4.56 (1.95)	6.48 (2.08)	0.001	0.90
Communication Skills	5.69 (1.17)	6.88 (1.06)	0.000	1.02
Quality of Communication	5.38 (1.32)	6.68 (1.27)	0.000	0.97
Self-Confidence	4.68 (1.62)	6.70 (1.24)	0.000	1.22
Theoretical Knowledge	3.55 (1.67)	7.88 (1.28)	0.000	2.87
Application of Knowledge	3.94 (1.76)	6.83 (1.33)	0.000	1.82

*t-test for dependent variables

^a^Effect sizes by Glass’s Δ

All questionnaires were developed according to the questionnaires which had been used in the evaluation of other CST [17, 34, 35], as they had proven to be practical, with modifications to fit the content of the current study.

**Fig. 1:**
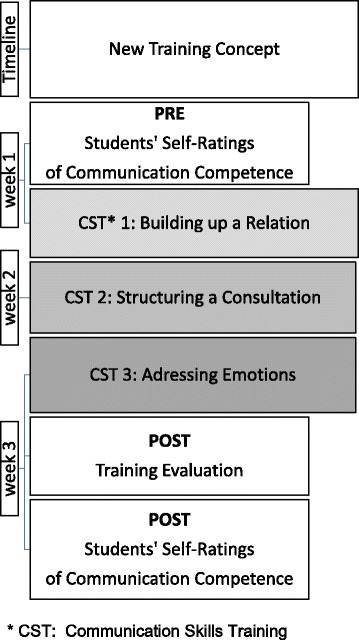
Study Design

The chronicle outline of the CST and collection of data are presented in Fig. 1.

### Statistical analysis

The data on acceptance were analyzed for mean and standard deviation (SD). Answers to forced choice questions were analyzed by giving the first choice a weight of one and the last choice a weight of nine and then computing the means of each item. Items were ranked in ascending order based on the mean rank generated by each item. A *t*-test was performed to calculate the changes in pre-post student self-ratings of competency. Additionally, we analyzed effect size by Glass’s ∆.

## Results

### Sample description

The study participants were 34 medical students, 82 % female (*n* = 28) and 18 % (*n* = 6) male, with an average age of 21.4 years (SD = 1.0). At the time of the study, all participants were in their first clinical year.

### Acceptance of CST

The response rate for the questionnaires was 100 %. As Table 1 shows, the acceptance of the general/overall didactics showed very good to good results, ranging from sufficient interaction (*M* = 1.15, *SD* = 0.44) to interesting didactic format (*M* = 1.76, *SD* = 0.86). The overall impression was good (*M* = 1.56 *SD* = 0.71), especially for the ‘interaction in the teaching groups’ (*M* = 1.15, *SD* = 0.44) and the ‘learning atmosphere’ (*M* = 1.18, *SD* = 0.39), which showed very good results. Quite similar results were found for the specific didactic elements: *M* = 1.12, *SD* = 0.33 for ‘feedback from the SPs’ and *M* = 2.32, *SD* = 0.77 for ‘theoretical introduction’. Interestingly, the didactic tools ‘practical training with SPs’ (*M* = 1.18, *SD =* 0.46) and ‘feedback of the SPs’ (*M* = 1.12, *SD =* 0.33) were rated highest. The ‘theoretical introduction’ (*M* = 2.32, *SD =* 0.77) and ‘memory card’ (*M* = 2.15, *SD =* 1.18) were the only items rated between satisfactory and good on average.

Again, the forced choice ranking underlines, how important participants evaluations were in the practical training with SPs (rank 1) and the various feedback elements (ranks 2–4) led by feedback from SPs (rank 2). Theoretical elements were at the bottom of the list, ranking 7 for the “theoretical introduction” and 9 for the ‘memory card’. However, the written summary of feedback, provided on the personal feedback form ranked low, at rank 8 see also Table 2.

### Changes in student feelings of communication competence

A further aim of the study was to investigate students’ self-ratings of communication competence before and after the new training, assessed by a 10 cm VAS (Table 3).

Five of the six key communication aspects that showed significant changes with effect sizes between *ES* = 0.52 and *ES* = 1.94. Only the item ‘patient’s perception’ did not change significantly (*p* = 0.125, *ES* = 0.30). The biggest improvement was found for ‘start of conversation’ (*p* < 0.0000) with a large effect size of *ES* = 1.94. For the general themes, all four improved significantly and showed large to very large effects (ES = 0.97–2.87). Especially, in the items ‘theoretical knowledge’ and ‘application of knowledge’, significant improvement was noted by the students (both *p* < 0.000) with *ES =* 2.87 and *ES* = 1.82.

## Discussion

In this study, we investigated medical students’ evaluations of a communication skills integrating specific and behavior-orientated feedback. This report presents the course structure and course content. Furthermore, we provide data on the acceptance and changes in students evaluations of their communication competence according to level one and two of Kirkpatrick’s pyramid of evaluation [32]. The main findings of this analysis are a high acceptance by students and significant improvements in students’ feelings about their communication competencies. The integrated 360° feedback was highly accepted and appreciated by the students.

The acceptance clearly shows that two elements, i. e. practical training and feedback, are crucial to the training concept. In the analysis of the different types of our feedback concept, feedback from SPs was rated best, followed by feedback from the trainer and colleagues. These results were assessed by evaluation and by a forced choice ranking. Only the written summary of feedback was evaluated as less than good. Perhaps students did not perceive an additional benefit to that tool. However, the good evaluation of feedback by SP points to the powerful tool of an SP not only in simulating reality but also in providing professional and specific feedback. This is consistent with Bokken et al. [36], who note that students appreciate feedback by SPs, especially when practicing communication skills.

Our training significantly increased students’ self-ratings of competence in specific communication aspects with large effect sizes in five out of six aspects of communication and also in the global rating. In particular, the aspect of starting a conversation improved the most.

Students’ self-ratings of communication competence in overall communication show great development in all aspects: the greatest effects were observed in the application of knowledge and theoretical knowledge. Whereas the former seems obvious since the main focus of the training is on the practical application of communication skills, the highest effect size for theoretical knowledge in a skill course seems slightly surprising, especially after the students’ evaluation wherein they expressed that they did not see much benefit in the theoretical part of the training. We could assume that practical and feedback elements outshined the theoretical introduction, which was rated the lowest. However, it might also be the case that students internalized the theoretical aspects for the first time by practicing and receiving specific feedback, and therefore, showed a significant increase in their theoretical knowledge.

Our concept builds on and integrates aspects of the current literature on feedback in education [7], particularly in clinical settings [2–4, 9–12, 20, 21, 28]. Some of the results may be reflected in the current literature: for example, Molley and Boud [24] note the importance of a good framework for receiving feedback from the student side and specify the attributed expertise of the feedback provider by the student, a constructive and respectful learning atmosphere, feedback and learning motivation. In our concept, we place great value on a constructive learning atmosphere by reinforcing good behavior and finding constructive alternatives to improvable behavior. This approach was evaluated by our students as having one of the highest ranks (*M* = 1.8; *SD* = 0. 39). Boehler et al. [23] investigated learning outcomes and the perceptions of different kinds of feedback. They found that students preferred praise over constructive behavior-orientated feedback, but the results of the instant study diverge from that findings as the students in this study evaluated feedback and gave it the highest ranks in our forced choice question after the practical training.

These results agree with the conclusions by Watling and Lingard [37], who discussed different aspects on how perceivers may accept personal feedback: It seems that the constructive approach in a respectful atmosphere was also key in our concept.

As our training time was shorter than recommended by experts [13], we sought to maximize the effects of our training as much as possible by optimizing the feedback process using the 360° approach. Findings indicate that, besides the great acceptance of our concept, seems to have been highly efficient increasing a sense of self competence. In the next step, we will have to investigate objective behavioral efficacy.

### Strengths and limitations

The strengths of our study are the successful integration of behavior-orientated 360° feedback in a routine communication skills training class offered in a current medical curriculum; therefore, its feasibility in everyday teaching seems to be assured.

We asked 34 students to volunteer in this study. They were informed that it was a study, and consent was acquired from each participant to take part in testing a teaching concept, but participants were blind with any details of the content.

We collected data from only one cohort in the winter term 2014/15 as part of the tested program. Repeated testing in other cohorts and in different centers is needed to confirm the findings.

Our evaluation form built on other frequently used evaluation questionnaires [3, 17], and produced strong face validity; however it has not been validated further. The questionnaire for assessing self-competence has previously proven to be change sensitive [17, 18].

## Conclusions

Our findings support the notion that specific and behavioral-oriented feedback in the context of undergraduate CST is well accepted by students. It enhances the learning success by promoting positive changes in student feelings of communication competence. Even from a trainer’s or SP’s point of view, this specific and behavioral, feed-forward approach may help produce a didactical structure that leads to a constructive learning environment. This well accepted method may easily be transferred to other practices or settings. Further research should evaluate the impact of this training on behavioral outcomes and, therefore, on the objective communication performance of the students.

## Additional files

Additional file 1: Memory Card: Key points of good communication. (DOCX 38 kb)
Additional file 2: Evaluation questionnaire of the training course. (DOCX 45 kb)
Additional file 3: Communication Skills Training – pre-Assessment Self-Rating of Communication Competence before the training course. (DOCX 569 kb)
Additional file 4: Communication Skills Training Post-Assessment: Self-Rating of communication Competence after attending the training (3 × 1.5 h). (DOCX 565 kb)

## Abbreviations

CST: Communication skills training
FB: Feedback
ES: Effect sizes
M: Mean
p: Significance
SD: Standard deviation
SP: Standardized patients
VAS: Visual Analogue Scale
