# Supplementary material for: Integrating 360° behavior-orientated feedback in communication skills training for medical undergraduates: concept, acceptance and students’ self-ratings of communication competence
Source: BMC Med Educ. 2016 Oct 18;16:271. doi: 10.1186/s12909-016-0792-0 (PMC5069808; doi:10.1186/s12909-016-0792-0)
Supplement: Additional file 1: — Memory Card: Key points of good communication. (DOCX 38 kb) [file 12909_2016_792_MOESM1_ESM.docx]

**MEMORY CARD**

**A Start of Conversation**

**A1** Initiate the conversation appropriately

**A2** Get an idea of the patient´s perception

**B Structure of Conversation**

**B1** Actively give structure to the conversation (set an agenda)

**B2** Set sub-sections

**C Patient´s Emotions**

**C1** Recognize the patient´s emotions and name them

**C2** Offer emotional support

**MEMORY CARD**

**D End of Conversation**

**D1** Summarize the content of the conversation and close appropriately

**E Communication Skills**

**E1** Use clear and appropriate words

**E2** Use appropriate non-verbal communication

**E3** Adjust your pace and make appropriate pauses

**E4** Offer the patient the chance to ask questions

**E5** Check whether the patient has understood the consultation
