# Supplementary material for: Integrating 360° behavior-orientated feedback in communication skills training for medical undergraduates: concept, acceptance and students’ self-ratings of communication competence
Source: BMC Med Educ. 2016 Oct 18;16:271. doi: 10.1186/s12909-016-0792-0 (PMC5069808; doi:10.1186/s12909-016-0792-0)
Supplement: Additional file 2: — Evaluation questionnaire of the training course. (DOCX 45 kb) [file 12909_2016_792_MOESM2_ESM.docx]

**Anonymisation Code**:

Month and year of your birthday (MM.YY) ☐☐☐☐

Your mother´s initials ☐☐

Todays date (DD.MM.YY) ☐☐☐☐☐☐

**Group-No.: [IG]**

**Please evaluate how you have experienced the Communication Skills Trainings 1 – 3 regarding the general didactics.**

__________________________________________________________________________

very good very poor

Practical relevance of topics 1 2 3 4 5 6

Practice orientation 1 2 3 4 5 6

Interesting didactic conditioning 1 2 3 4 5 6

Sufficient interaction 1 2 3 4 5 6

Constructive learning atmosphere 1 2 3 4 5 6

### Personal profit 1 2 3 4 5 6

###

### Fulfilled expectations 1 2 3 4 5 6

### Overall impression 1 2 3 4 5 6

______________________________________________________________________

## How do you rate the specific didactic elements?

very good very poor

Theoretical introduction 1 2 3 4 5 6

Memory card 1 2 3 4 5 6

Practical training with standardised patients 1 2 3 4 5 6

Monitoring tasks 1 2 3 4 5 6

Self-reflection 1 2 3 4 5 6

Feedback from colleagues 1 2 3 4 5 6

very good very poor

Feedback from standardised patients 1 2 3 4 5 6

Feedback from trainer 1 2 3 4 5 6

Personal feedback form 1 2 3 4 5 6

**Please rank the 9 above-mentioned didactic elements putting**

**1 for the most important priority and**

**9 for the less relevant priority.**

**Please do not give elements the same weighting (forced choice).**

☐ Theoretical introduction

☐ Memory card

☐ Practical training with standardised patients

☐ Monitoring tasks

☐ Self-reflection

☐ Feedback from colleagues

☐ Feedback from standardised patients

☐ Feedback from trainer

☐ Personal feedback form

**Which personal strengths in communication have been fed back to you in the training?**

**What can you do better in the future regarding your communication skills?**

**Anonymisation Code**:

Month and year of your birthday (MM.YY) ☐☐☐☐

Your mother´s initials ☐☐

Todays date (DD.MM.YY) ☐☐☐☐☐☐

**Group-No.: xx**

### What did you like most about the training?

What in your view we can do to **improve the training** for the next time:

**We thank you for your cooperation!**
