# Supplementary material for: Integrating 360° behavior-orientated feedback in communication skills training for medical undergraduates: concept, acceptance and students’ self-ratings of communication competence
Source: BMC Med Educ. 2016 Oct 18;16:271. doi: 10.1186/s12909-016-0792-0 (PMC5069808; doi:10.1186/s12909-016-0792-0)
Supplement: Additional file 3: — Communication Skills Training – pre-Assessment Self-Rating of Communication Competence before the training course. (DOCX 569 kb) [file 12909_2016_792_MOESM3_ESM.docx]

**Communication Skills Training - Pre-Assessment**

Participating medical undergraduates

**Self-Rating of Communication Competence**

Dear participants of the research project

**Didactic Optimisation of Communication Skills Training** – a randomised-controlled trial in the field of Medical Didactics,

in the following we want you to provide some information regarding your previous ‘doctor-patient-conversations´.

Please answer the questions completely and truthfully.

Data will be treated as strictly confidential. According to data protection regulations informations you provide will be evaluated anonymously and used only for research purposes.

**The following example explains to you how the questions should be answered:**

After each statement there is a 10 cm line indicating a continuum between two extremes, e.g. ‘I agree’ to ‘I disagree’. Please answer the question drawing a vertical stroke on the given line: The closer the stroke is to ‘I agree’ the stronger you go along with the statement; the closer it is to ‘I disagree’ the stronger your dissent about that phrase. We are interested in your subjective assessment.

In case, you want to make corrections, e.g. because you made the stroke at the wrong place, we ask you – as seen in the following sample – to completely colour the ‘wrong’ stroke and to set another one at the correct place.


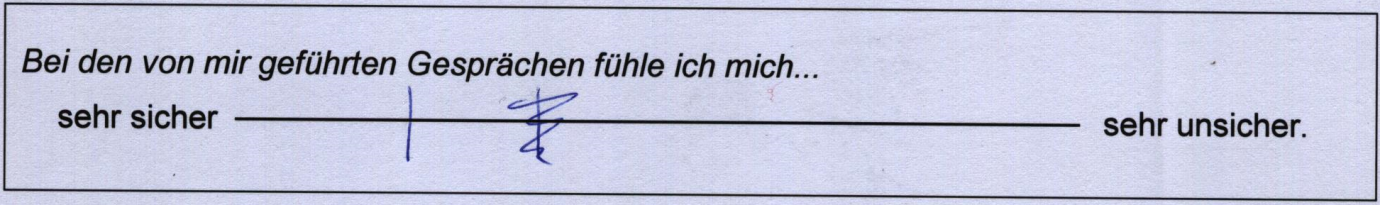


**Anonymisation Code**:

Month and year of your birthday (MM.YY) ☐☐☐☐

Your mother’s initials ☐☐

Today’s date (DD.MM.YY) ☐☐☐☐☐☐

**Group-No.: [IG/CG]**

**How do you experience your previous ‘doctor-patient-conversations’ in general?**

*I feel*

_A1_ very safe very unsafe

*about how to* ***start a conversation****.*

*I find it*

_A2_ very easy very hard

*to assess* ***patient´s perception****.*

*I find it*

_B1*_ very easy very hard

*to formulate a shared main conversation topic.*

*I find it*

_B2*_ very easy very hard

*to set subtopics within the conversation.*

*In talks hold by myself I can recognise patient´s emotions*

_C1*_ very well very poorly.

*In talks hold by myself I can emotionally support patients*

_C2*_ very well very poorly.

*I find it*

_E1*_ very easy very hard

*to assess if the patient understands the wording I use.*

**Please turn round!**

*I find it*

_E2*_ very easy very hard

*to assess if my non-verbal communication reaches the patient.*

*I find it*

_E3*_ very easy very hard

*to assess if and when within the talk pauses are adequate.*

*I find it*

_E4*_ very easy very hard

*to assess if the patient could ask all the questions wanted.*

*I find it*

_E5*_ very easy very hard

*to assess if the patient has understood the previous points discussed.*

*I feel*

_D1_ very safe very unsafe

*about how to* *set an* ***end of conversation****.*

***Global****ly, I rate my communication competence as*

_F1_ very good very poor.

*I rate the quality of communication within the talks hold by myself as*

very good very poor.

*Within the talks hold by myself I feel*

very **self-confident** very self-inconfident.

*I have*

very good very poor

***theoretical knowledge*** *about how to conduce conversations best according to actual standards.*

*I can* ***apply my knowledge*** *in concrete settings*

very well very poorly.

**Thank you for completing this report!**

* Statistical Sub-Scales:

B1/B2 = Structure of Conversation; C1/C2 = Patient´s Emotions; E1- E5 = Communication Skills
